# Supplementary material for: Use of the Journal Impact Factor in academic review, promotion, and tenure evaluations
Source: eLife. 2019 Jul 31;8:e47338. doi: 10.7554/eLife.47338 (PMC6668985; doi:10.7554/eLife.47338)
Supplement: Supplementary file 1. [file elife-47338-supp1.pdf]

Supplemental information: Mentions of the Journal Impact Factor in review, promotion, and tenure documents

Erin C. McKiernan, Lesley A. Schimanski, Carol Muñoz Nieves, Lisa Matthias, Meredith T. Niles, Juan Pablo Alperin

Our goal with this supplemental document is to provide a list and a qualitative analysis of each of the mentions (excluding most repetitions) of the Journal Impact Factor (JIF), or closely related terms, found in our representative sample of RPT documents.

In the main manuscript, we provide details on how we arrived at the different terms in our coding terminology. Briefly, we used QSR International's NVivo software to search the documents for the following terms or phrases (singular and plural): (1) 'impact factor', (2) 'impact score', (3) 'impact metric', (4) 'impact index', (5) 'high(-)impact journal', (6) 'impact of the journal', and (7) 'journal('s) impact'. The results of each text query were placed in an NVivo "node" that contained the text surrounding each of the mentions. We then exported the content of each node. In some cases, the software extracted complete sentences, while in other cases it pulled only fragments and we retrieved the rest of the text manually to provide better context.

We classified each of the mentions of the JIF as supportive, cautious, or neutral. We also analyzed the mentions to see if the JIF was associated with concepts such as quality, prestige, and importance. See the main manuscript for more details. Here, we have color coded terms of interest for emphasis and ease of identification. However, it should be noted that just because a word is highlighted does not mean the mention was coded as making an association between that concept and the JIF (our coding was stricter). Qualitative assessments can be found below each mention. The color coding scheme is as follows:

- **blue**: terms from the coding terminology
- **red**: term 'quality'
- **magenta**: terms such as 'prestige/prestigious', 'reputation/reputable', and 'status/stature' (referring to publications and/or publication venues)
- **orange**: terms such as 'impact' (outside the coding terminology), 'importance', and 'significance' (referring to publications and/or research)
- **green**: statements which question or discourage use of the JIF

Contents

1 Arizona State University **6**

1.1 School of Mathematical and Natural Sciences 6

2 Boise State University **7**

2.1 Guidelines for Promotion and Tenure (2017), Department of Psychological Science, College of Arts and Sciences 7

2.2 Tenure and Promotion Policy (2017), College of Arts and Sciences 7

3 John Carroll University **8**

3.1 Tenure and Promotion to Rank of Associate, Policies and Procedures (2015), Department of Counseling 8

4 Loyola University Chicago **9**

4.1 Tenure and Promotion Guidelines and Procedure (2012), Environmental Science Department 9

4.2 Tenure and Promotion Guidelines (2015), Institute of Environmental Sustainability 9

5 McMaster University **11**

5.1 Tenure, Permanence and Promotion: A Guide for Chairs and Directors (Workshop April 2017) 11

6 Memorial University of Newfoundland **12**

6.1 Promotion and Tenure, Non-Bargaining Unit Criteria for Clinical Faculty (2013), Faculty of Medicine 12

7 Ohio University – Main Campus **13**

7.1 Minimal Criteria for Promotion and Tenure (2015), Russ College of Engineering and Technology 13

7.2 Promotion and Tenure Policy (2014), College of Health Sciences and Professions 14

8 Pace University **15**

8.1 Guidelines for the Preparation and Evaluation of Tenure and Promotion Dossiers (date of creation not specified, but goes into effect 2020) 15

9 Purdue University **16**

9.1 Criteria for Promotion to the Ranks of Associate Professor With Tenure and Full Professor (2014), Department of Anthropology 16

9.2 Guideline for Academic Promotion and Tenure in School of Health Sciences (2012), School of Health Sciences 16

10 San Diego State University **19**

10.1 RTP Policies and Procedures AY 2014-15, College of Education 19

10.2 Reappointment, Tenure, and Promotion Handbook For Candidates (2016-2017) . . 20

11 Simon Fraser University **22**
11.1 Criteria for Promotion (year not specified), Department of Psychology 22
11.2 Renewal, Tenure & Promotion Workshop (April 2016) 23

12 Texas A & M University-Corpus Christi **24**
12.1 Faculty Handbook (2012), Faculty of Science 24

13 University of Alberta **25**
13.1 Criteria for Merit Increments, Tenure and Promotion (2012), Faculty of Science . . . 25
13.2 Guidelines for the Evaluation of Academic Staff for Merit Increments, Tenure, and Promotion (2007), Faculty of Medicine and Dentistry 25

14 University of British Columbia **28**
14.1 Annotated CV, Professoriate Stream (2016), Faculty of Medicine 28

15 University of Calgary **29**
15.1 Guidelines for Appointment, Promotion, and Tenure of Academic Staff (2008), Faculty of Veterinary Medicine 29

16 University of California, Los Angeles **30**
16.1 Criteria for Promotion to Tenure (year not specified), Department of History 30

17 University of California, San Diego **31**
17.1 Where CAP Stood, 2015-16, Committee on Academic Personnel 31

18 University of Central Florida **32**
18.1 Promotion and Tenure Criteria (2016), Department of Marketing, College of Business Administration 32
18.2 Policies and Procedures for Promotion and Tenure (2014), Department of Communication Sciences and Disorders, College of Health & Public Affairs 32
18.3 Criteria for Promotion and Tenure (2014), School of Social Work, College of Health & Public Affairs 33
18.4 Department Criteria for Tenure and Promotion (2016), Department of Anthropology, College of Sciences 33
18.5 Guidelines for Promotion and Tenure (2012), Department of Biology, College of Sciences 34
18.6 Departmental Criteria for Tenure and Promotion (2011), Department of Political Science, College of Sciences 34
18.7 Department Criteria for Tenure and Promotion (2015), Department of Sociology, College of Sciences 35
18.8 Promotion and Tenure Guidelines (2013), Department of Statistics, College of Sciences 35

19 University of Guelph **36**
19.1 Department Guidelines Document for Tenure, Promotion, and Performance Assessment (2012), Department of Psychology, College of Social & Applied Human Sciences . . 36

19.2 Guidelines for Tenure, Promotion and Performance Assessment (2008), Department of Human Health and Nutritional Sciences 37

20 University of La Verne **38**

20.1 Policies and Procedures on Annual, Probationary Status, Promotion, and Tenure Reviews (2016), Department of Psychology 38

21 University of North Carolina at Chapel Hill **39**

21.1 Policy Manual for Faculty Appointments, Promotions, and Tenure, School of Education 39

21.2 Guidelines for Appointment, Reappointment and Promotion of Faculty (2014), School of Medicine 39

21.3 Personnel Promotion and Tenure Procedures (2013), School of Social Work 40

22 University of North Carolina at Greensboro **41**

22.1 Best Practices in Tenure and Promotion (2016), College of Arts & Sciences 41

22.2 Promotion and Tenure Guidelines (2013), Department of Nanoscience, Joint School of Nanoscience and Nanoengineering 41

22.3 General Evaluation Guidelines for Reappointment, Promotion and Tenure, and Post-Tenure Review (2014), Department of Nutrition, School of Health and Human Sciences 42

23 University of North Dakota **43**

23.1 Faculty Handbook (2008), Department of Biology 43

24 University of Saskatchewan **44**

24.1 Standards and Criteria Used by the Food and Bioproduct Sciences Departmental Salary Committee (DSC) to Award Special Increases (SI) to Faculty Members (2012), Department of Food & Bioproducts, College of Agriculture & Bioresources 44

24.2 Standards for Promotion and Tenure, College of Medicine (2011) 44

24.3 Guidelines for Preparation of Case Files for Renewal of Probation, Tenure and Promotion (2016) 45

24.4 Criteria and Standards (2003), College of Arts and Science, Department of Physics and Engineering Physics 45

25 University of Southern Mississippi **46**

25.1 Department Guidelines for Tenure and Promotion (2016), Department of Political Science, International Development, and International Affairs 46

25.2 Promotion and Tenure Guidelines (2011), Department of Educational Studies and Research 46

25.3 Revision to Tenure and Promotion Documentation (2015) 47

25.4 Promotion and Tenure Standards, Department of Philosophy and Religion, University of Southern Mississippi 47

26 University of Utah **48**

26.1 Policies and Procedures for Retention, Promotion, and Tenure of Regular Faculty (2012), Department Of Political Science, College of Social & Behavioural Science 48
26.2 Policies and Procedures for Retention, Promotion, and Tenure Procedures and Criteria (2011), Department of Sociology, College of Social & Behavioral Sciences 49

27 University of Vermont **50**
27.1 Guidelines for Reappointment, Promotion, and Tenure (2009), College of Agriculture and Life Sciences 50
27.2 Statement Regarding the Appointment, Tenure, and Promotion of Tenure-Track Faculty (2015), College of Arts and Sciences 50
27.3 Guidelines for the C.V. for Green Sheets (2013) 51
27.4 Reappointment, Promotion and Tenure (RPT) Guidelines and Forms, Green Sheet Form (2016) 51

28 University of Victoria **52**
28.1 Faculty Evaluation Policy (2016), Faculty of Science 52

29 University of Windsor **53**
29.1 Standards: Example of multiple minimum thresholds in one document (2016) 53
29.2 Sample Research Evaluation Rubric (2016) 54
29.3 Working Session on Developing Promotion and Tenure Criteria for Research (2016) 55

30 University of Wisconsin-Madison **56**
30.1 Guidelines for Recommendations for Promotion or Appointment to Tenure (2014), Faculty Division of the Physical Sciences 56
30.2 Format for Tenure Dossiers submitted to the Physical Sciences Divisional Committee, Checklist for Departments (2014) 56
30.3 Getting Promoted From Assistant to Associate Professor on the Tenure Track (2015), Faculty Division of Biological Sciences, Department of Radiology 57

1 Arizona State University

1.1 School of Mathematical and Natural Sciences

> Publications possess relative merit and it is the responsibility of the faculty member to provide an estimation of this merit using the following criteria: relative contribution of the faculty member for coauthored manuscripts (e.g., a percentage value representing overall proportional contribution to the finished product); **quality** of the journal (both common knowledge and weighting factors, such as **impact indices**); and the **impact** of the contribution to the discipline.

Sentiment: Supportive
Claims to measure: Quality

> Three levels of scholarly contribution **quality** are recognized: A, B, and C. These three levels provide a means by which the **quality** of contributions can be ranked in addition to quantity (e.g., publication rate and total number of contributions) in assigning annual review scores. In multiauthored endeavors, the faculty member should state the significance of his/her contribution to the project. For all items, the faculty member should state the appropriate level (see below) with a brief explanation of this choice (e.g., **impact factor of journal**, refereed publication with an undergraduate, etc.)

Sentiment: Supportive
Claims to measure: Quality

2 Boise State University

2.1 Guidelines for Promotion and Tenure (2017), Department of Psychological Science, College of Arts and Sciences

> Although quantity of scholarship is certainly important, **quality** is also important. In addition to meeting quantitative requirements in the area of scholarship, successful candidates for tenure and promotion must make a compelling case for excellence in scholarship. Examples of evidence related to **quality** include the type of outlet (e.g., **quality** of journals), **impact** on the field (e.g., citations and **journal impact**), and/or **impact** on the greater community for technical reports (e.g., audience of the outlet, such as a small private business versus a major Government agency).

Sentiment: Supportive
Claims to measure: Quality and impact/importance/significance

2.2 Tenure and Promotion Policy (2017), College of Arts and Sciences

> If a department employs specific **metrics of impact** or **quality** for the purpose of evaluation relative to tenure and promotion (e.g., H-index or critical reviews), the processes and standards shall be described in departmental policy.

Sentiment: Neutral
Claims to measure: Unspecified

3 John Carroll University

3.1 Tenure and Promotion to Rank of Associate, Policies and Procedures (2015), Department of Counseling

> Publishing articles in refereed journals is considered the strongest type of publication. The **reputation** of the journal, the acceptance rate and **impact scores** are important factors when evaluating someone's accomplishment in this category.

Sentiment: Supportive
Claims to measure: Unspecified

> Information/Evidence to support the **quality** and **significance** of candidate's published and presented research
> ...
> - Acceptance rates, readership/circulation, **impact scores** and/or information about the professional organization/conference to establish the **importance/significance** of candidate's work to the profession

Sentiment: Supportive
Claims to measure: Quality and impact/importance/significance

4 Loyola University Chicago

4.1 Tenure and Promotion Guidelines and Procedure (2012), Environmental Science Department

> "For promotion to Full Professor, publication rates should average one manuscript per year over the five years preceding promotion. The length of articles and scientific **significance**, as measured by citations and **journal impact**, may also be considered, as will authorship on contributions to other scholarly works (e.g., reference and text books)."

Sentiment: Supportive
Claims to measure: Impact/importance/significance

4.2 Tenure and Promotion Guidelines (2015), Institute of Environmental Sustainability

> "Evaluation of a candidate's research will consider the **quality** of the work, as well as the quantity of published products and the degree to which the candidates' research is original and advances the field. Web-based reference services, such as The Web of Science, will be used to quantify how many times the candidate's publications have been cited by others, and the **quality** of the journals will be assessed by their average **impact factors**."

Sentiment: Supportive
Claims to measure: Quality

4 Loyola University Chicago (continued)

4.2 Tenure and Promotion Guidelines (2015), Institute of Environmental Sustainability (continued)

> Candidates should have at least four manuscripts in peer-reviewed journals published or in-press in the five years preceding application for tenure and promotion to Associate Professor. The length of articles and scientific **significance**, as measured by citations and **journal impact factor**, will also be considered, as will authorship on contributions to other scholarly works (e.g., reference and text books).

Sentiment: Supportive
Claims to measure: Impact/importance/significance

> For promotion to Professor, the candidate must have an average publication rate of at least one article per year published in peer-reviewed journals in the five-year period preceding the application for promotion. These articles should be regularly cited by other researchers in the field. We will consider both the **quality** of the journal (as measured by the **journal's impact factor, or JIF**) as well as the number of citations of each publication. We will employ the metric: Article Impact Factor (AIF) = (**JIF** * citations) where "citations" represents the number of citations for the particular publication. Employing this metric, faculty have incentive to publish in the highest **quality** journals (which will increase the **JIF**) and simultaneously produce the highest **quality** research manuscripts, potentially increasing the number of citations, and increasing the AIF.

Sentiment: Supportive
Claims to measure: Quality

5 McMaster University

5.1 Tenure, Permanence and Promotion: A Guide for Chairs and Directors (Workshop April 2017)

> “
> - Encourage to submit research to peer-review process as soon as possible.
>   - Time lags for submission, editorial responses, acceptance and publication.
> - **Impact factors** and rejection rates
>
> ...
> ”

Sentiment: Neutral
Claims to measure: Unspecified

6 Memorial University of Newfoundland

6.1 Promotion and Tenure, Non-Bargaining Unit Criteria for Clinical Faculty (2013), Faculty of Medicine

> APPENDIX II - SCHOLARSHIP IN DISCOVERY
> Criteria (professor)
> ...
>
> - Substantial record of research productivity with either a landmark paper in a **prestigious** international journal or multiple papers in **high-impact journals** that make a **significant impact** on the field

Sentiment: Supportive
Claims to measure: Impact/importance/significance

> APPENDIX II - SCHOLARSHIP IN DISCOVERY
> Criteria (professor)
> ...
>
> - Chair or Deputy Chair of a grant review panel or editor of a **high-impact journal**

[Note: A similar quote is found in the same document under criteria for ‘Excellence in Leadership’]

Sentiment: Supportive
Claims to measure: Unspecified

7 Ohio University – Main Campus

7.1 Minimal Criteria for Promotion and Tenure (2015), Russ College of Engineering and Technology

> "The Russ College of Engineering and Technology has established metrics for the evaluation of departments, programs and faculty, and for establishing college workload policy. These serve as guidelines for those making promotion and tenure decisions in the college but candidates must recognize that other factors are taken into account as well. For example, the **quality** and **impact** of publications must be considered as well as the quantity. Specifically, the **quality**/**reputation** of a given journal or refereed conference (e.g., **impact factor**) will be considered along with **impact** of a given paper (e.g., citation count)."

Sentiment: Supportive
Claims to measure: Quality and prestige/reputation/status

> "List relevant peer-reviewed journal and conference papers published over the last five years (or since last promotion or initial appointment, whichever is less) related to pedagogy or other relevant areas of education. Include the **journal's impact factor** (or equivalent journal ranking data) and the number of citations of the article(s)."

Sentiment: Supportive
Claims to measure: Unspecified

7 Ohio University – Main Campus (continued)

7.1 Minimal Criteria for Promotion and Tenure (2015), Russ College of Engineering and Technology (continued)

> List title, co-authors, journal, volume number, date, page numbers, etc., of articles that have been published or have been accepted for publication but not yet published. Include the **journal's impact factor** (as given by the Journal Citation Reports from the Web of Science) or equivalent journal ranking (must list source of ranking) and the number of citations of the article(s).

Sentiment: Supportive
Claims to measure: Unspecified

7.2 Promotion and Tenure Policy (2014), College of Health Sciences and Professions

> Quantity is neither the primary nor the driving factor in assessing scholarly/creative activity. Schools and departments may choose to identify various quantitative markers, and it is a combination of quantity and **quality** of scholarship that is ultimately assessed in evaluating the candidate. The **quality** of the work, influence the work produces, and the level of contribution to a particular body of knowledge are the substantive issues that will be reviewed for promotion and/or tenure. Markers of **quality** of publications may include **impact factors of journals**, number of citations of published work, and audience of journal.

[Note: The above quote is first found under Group I Faculty, and is repeated in the same document under Clinical Faculty.]

Sentiment: Supportive
Claims to measure: Quality

8 Pace University

8.1 Guidelines for the Preparation and Evaluation of Tenure and Promotion Dossiers (date of creation not specified, but goes into effect 2020)

> Publications and **quality** of journals: For excellence in research, candidates should demonstrate their ability to publish their work in **reputable** journals, scholarly anthologies or collections, and conference proceedings. Refereed publications, and/or peer-reviewed journals, with a high **impact factor** carry special weight. For books and monographs, the **quality** of the press is important. Similarly, creative activities at recognized venues are highly regarded.

Sentiment: Supportive
Claims to measure: Quality and prestige/reputation/status

> Some suggested subheadings for appendices include:
> ...
> **Impact Factor** and Citation Reports. Include citation of your work, (e.g. by other authors or speakers), **impact factor** and acceptance rate of journals your work appears.

Sentiment: Supportive
Claims to measure: Unspecified

9 Purdue University

9.1 Criteria for Promotion to the Ranks of Associate Professor With Tenure and Full Professor (2014), Department of Anthropology

> The **quality** of a candidate's research will be an important component of the assessment of scholarly contribution. Members of the primary committee have the responsibility to examine the publications of candidates when making judgments of **quality**. In addition, the **quality** of the journals as indicated by such things as **reputation**, editorial board members, **impact factors**, and rejection rates will be considered in judgments of **quality**. The department expects that some of the candidate's publications will have appeared in major influential journals in anthropology or in the candidate's area of specialization.

Sentiment: Supportive
Claims to measure: Quality

9.2 Guideline for Academic Promotion and Tenure in School of Health Sciences (2012), School of Health Sciences

> Each candidate for promotion should have the opportunity to document his/her contributions as a scholar. This should include peer-reviewed and non-peer reviewed publications, scholarly presentations, and intellectual property development. Candidates should provide some measure of journal ranking or **impact factor** for each publication published during the period in rank.

Sentiment: Supportive
Claims to measure: Unspecified

9 Purdue University (continued)

9.2 Guideline for Academic Promotion and Tenure in School of Health Sciences (2012), School of Health Sciences (continued)

> "The candidate should publish at least 3 peer-reviewed publications each year, or less than 3 but in **high impact journals**, prior to promotion. The candidate for Full Professor should show the continuity of a strong publication record, at least 3-4 peer-reviewed publications per year in **high impact journals**, since the candidate's promotion to the associate professor rank."

Sentiment: Supportive
Claims to measure: Unspecified

> "While quoting the Scientific Citation Index of each paper is not mandatory, publications in **high impact journals** covering the candidate's own research field is expected. Candidates are encouraged to provide information for evaluating the **quality** of the journals in which their work is published. This may include **impact factors** (relative to other publications in the field), rejection rates, editorial board membership, and overall ranking of the journal in the field. This information will be combined with the judgments of outside reviewers in evaluating the **quality** of the candidate's research scholarship."

Sentiment: Supportive
Claims to measure: Quality

9 Purdue University (continued)

9.2 Guideline for Academic Promotion and Tenure in School of Health Sciences (2012), School of Health Sciences (continued)

> The Instructions say in Section B.1.d. that "It would be helpful to include where the publications [of a candidate] are ranked in one's field (first tier, second tier, third tier)." In some fields, this kind of information is usually provided by listing the **impact factor for the journal** in which a publication appeared. When **impact factors** are listed, it is useful to include some comment in the document about what values of **impact factors** should be considered as high, medium, or low for a candidate's field.

Sentiment: Supportive
Claims to measure: Unspecified

10 San Diego State University

10.1 RTP Policies and Procedures AY 2014-15, College of Education

> "Candidate provides supporting materials to validate any references to acceptance rates of journals, reviews of scholarly work, **journal impact factors**, number of citations of published work, or other specifics on teaching, service or professional growth mentioned in their statement."

[Note: This quote is found twice in the document in different sections.]

Sentiment: Neutral
Claims to measure: Unspecified

> "Evidence for evaluating professional growth, as identified and defined in department or school and college guidelines, shall comprise: externally reviewed professional growth activities including, as a primary and necessary element, refereed publications of merit...**Quality** of the evidence may be identified in several ways, appropriate to the various disciplines, and may include: published or unpublished reviews of a candidate's work; external reviews; number of citations for a published work; **journal impact factors**; acceptance rates; **stature** of journal or book editorial boards; and/or **reputation** of journal or publisher in the field."

Sentiment: Supportive
Claims to measure: Quality

10 San Diego State University (continued)

10.1 RTP Policies and Procedures AY 2014-15, College of Education (continued)

> Question: How looking at On-Line Journals, especially with many of these journals charging for publication? Edith: The Committees will have to discuss this, perhaps at the College level. We generally consider **impact factors** and acceptance rates.

Sentiment: Supportive
Claims to measure: Unspecified

10.2 Reappointment, Tenure, and Promotion Handbook For Candidates (2016-2017)

> Be aware of the **impact factor** or selectivity of the venues in which you publish, your status as an author in group-authored publications, and hierarchies of value in the different genres of scholarly activity in your field: for example, a peer reviewed article may be valued more than a conference presentation, book review, or encyclopedia article.

Sentiment: Supportive
Claims to measure: Unspecified

10 San Diego State University (continued)

10.2 Reappointment, Tenure, and Promotion Handbook For Candidates (2016-2017) (continued)

> Evidence for evaluating professional growth, as identified and defined in department / school or school and college guidelines, shall comprise: externally reviewed professional growth activities including, as a primary and necessary element, refereed publications of merit...**Quality** of the evidence may be identified in several ways, appropriate to the various disciplines, and may include: published or unpublished reviews of a candidate's work; external reviews; number of citations for a published work: **journal impact factors**; acceptance rates; **stature** of journal or book editorial boards; and/or **reputation** of journal or publisher in the field.

[Note: This same quote also appears in the RTP Policies and Procedures AY 2014-15 and the RTP Handbook For Reviewers at San Diego State University.]

Sentiment: Supportive
Claims to measure: Quality

11 Simon Fraser University

11.1 Criteria for Promotion (year not specified), Department of Psychology

> The TPC [Tenure and Promotion Committee] may consider a variety of indicators of success in scholarship. A non-exhaustive list of indicators of high-**impact** scholarship includes a **significant** body of research that has been published in appropriate peer-reviewed journals; non-peer-reviewed publications such as books and book chapters...University policy also requires evaluation of the candidate's record by external or internal referees of high academic stature; the referees' reports will be an important component of the Psychology TPC's overall evaluation. The TPC may additionally consider metrics such as citation figures, **impact factors**, or other such measures of the reach and **impact** of the candidate's scholarship.

Sentiment: Supportive
Claims to measure: Impact/importance/significance

> It is expected that the candidate will be productive in terms of generating refereed journal articles from his or her ongoing independent research program. No specified number of publications is required, but rather, emphasis will be placed on the **quality** of the publications, and **impact** on the field. Factors used to assess **quality** may include such measures as citations of publications, evaluations of the work by external referees, and the **impact factor of the journals** in which they are published.

Sentiment: Supportive
Claims to measure: Quality

11 Simon Fraser University (continued)

11.1 Criteria for Promotion (year not specified), Department of Psychology (continued)

> For promotion to Full Professor, the candidate will need to present evidence of an international **reputation** in his or her field. This will normally be assessed by establishing that there is a **significant** body of research that has been published in peer reviewed journals. In addition to evaluations solicited from external referees, the **quality** of this research may be assessed using the citation figures for publications and by examining the **impact factor of the journals** involved.

Sentiment: Supportive
Claims to measure: Quality

11.2 Renewal, Tenure & Promotion Workshop (April 2016)

> Most Common Problems
> TPC [Tenure and Promotion Committee] incorrectly counting/evaluating publications
> ...
> - Include citation data and **journal impact factors** if appropriate

Sentiment: Neutral
Claims to measure: Unspecified

12 Texas A & M University-Corpus Christi

12.1 Faculty Handbook (2012), Faculty of Science

> Publication within the discipline is considered the most important indication of scholarship...Primarily, these publications should provide evidence of the development of an independent research program within the discipline and especially in **high-impact journals**.

Sentiment: Supportive
Claims to measure: Unspecified

13 University of Alberta

13.1 Criteria for Merit Increments, Tenure and Promotion (2012), Faculty of Science

> Of all the criteria listed, the one used most extensively, and generally the most reliable, is the **quality** and quantity of published work in refereed venues of international **stature**. **Impact factors** and/or acceptance rates of refereed venues are useful measures of venue **quality**; however, it is the responsibility of the Chair of the Department to evaluate, through consultation with his/her colleagues and the use of expert opinion in the field, the **quality** of the research and scholarship of an individual.

Sentiment: Supportive
Claims to measure: Quality

13.2 Guidelines for the Evaluation of Academic Staff for Merit Increments, Tenure, and Promotion (2007), Faculty of Medicine and Dentistry

> Performance will be judged to be “Superior" when it exceeds expectations. There will be evidence of:
>
> - a substantial record of research productivity with either a landmark paper in a **prestigious** international journal or multiple papers in **high-impact journals** that make a **significant impact** on the field

Sentiment: Supportive
Claims to measure: Impact/importance/significance

14 University of Alberta (continued)

14.2 Guidelines for the Evaluation of Academic Staff for Merit Increments, Tenure, and Promotion (2007), Faculty of Medicine and Dentistry (continued)

> Examples of superior administrative contributions can be related to research or education and include contributions to the discipline such as serving as Chair of a grants panel or site visit, serving as editor of a **high-impact journal**, organizing a major national or international conference, or serving as president of a professional organization; recognition by receipt of a service award from a professional society would be considered meritorious.

Sentiment: Supportive
Claims to measure: Unspecified

> Performance will be judged to be "Superior" when it exceeds expectations. There will be evidence of:
> ...
> - exceptional administrative service such as serving as Chair or Deputy Chair of a grant review panel or editor of a **high-impact journal**

Sentiment: Supportive
Claims to measure: Unspecified

14 University of Alberta (continued)

14.2 Guidelines for the Evaluation of Academic Staff for Merit Increments, Tenure, and Promotion (2007), Faculty of Medicine and Dentistry (continued)

> "Criteria for superior performance in the research/scholarship category represent guidelines, not mandatory requirements (see C. Performance Standards). Superior performance will have been demonstrated if the candidate has published a substantial number of papers in good journals with some (or one landmark paper) making **significant** contributions to the field;...and/or has served as Chair of a grant review panel, editor of a **high impact journal** or organizer of a major national/international scientific or educational meeting."

Sentiment: Supportive
Claims to measure: Unspecified

14 University of British Columbia

14.1 Annotated CV, Professoriate Stream (2016), Faculty of Medicine

> Publications should be listed and numbered sequentially in chronological order starting with the earliest...Addition of other information, such as the **Journal Impact Factor**, Journal ranking in the field or the number of citations is optional and should be presented in parentheses at the end of the reference.

Sentiment: Neutral
Claims to measure: Unspecified

15 University of Calgary

15.1 Guidelines for Appointment, Promotion, and Tenure of Academic Staff (2008), Faculty of Veterinary Medicine

> Peer-Reviewed Publications: Special consideration is to be given to the **quality** of the publication and the nature of the authorship. Contributions of the applicant must be clearly documented. The **reputation** and **impact of the journal** or other publication format will be considered, but takes secondary consideration to the **quality** of the publication and the nature of the contributions. **Impact factors of journals should not be used as the sole or deciding criteria in assessing quality**. Similarly, citation rates shall be interpreted appropriately as relates to the discipline and/or area of research. Productivity should always be appropriate to the discipline and **quality** should always considered and not absolute numbers of publications.

Sentiment: Cautious
Claims to measure: Quality

16 University of California, Los Angeles

16.1 Criteria for Promotion to Tenure (year not specified), Department of History

> The CALL's concern that the candidate be "continuously and effectively engaged in creative activity of high **quality** and **significance**," should further be demonstrated through other publications that include peer reviewed articles in **high impact journals**, as well as evidence through submitted work in progress of new, or developing, research projects. The Committee realizes that there are fields in which the writing of peer reviewed articles may take precedence over the completion of a book, or book manuscript. In such cases the published work shall be judged for its creativity, originality, and high **impact** by the standards of that field, or sub-field, based on the careful evaluation of the candidate's published work by both departmental and external reviewers.

Sentiment: Supportive
Claims to measure: Quality and impact/importance/significance

17 University of California, San Diego

17.1 Where CAP Stood, 2015-16, Committee on Academic Personnel

> Candidates' research/creativity must have been what their department would expect for two review periods and there could be no weakness in teaching or service. The double publication norm is, of course, a signal and not a requirement, and CAP [Committee on Academic Personnel] routinely recommends accelerations if a candidate's publications are documented as especially **impactful**. **Quality** always is rewarded. In rare cases a candidate can receive an acceleration for extraordinary teaching and service and a publication record that is more than the regular merit level but not double. CAP welcomes data on journal acceptance rates and **impact factors**, citation rates and H-index, but **some CAP members (as do senior staff of scholarly societies) retain various degrees of skepticism about such measures**.

Sentiment: Cautious
Claims to measure: Unspecified

18 University of Central Florida

18.1 Promotion and Tenure Criteria (2016), Department of Marketing, College of Business Administration

> The research record should include publications in the best peer-reviewed journals in marketing, as evidenced by **impact factors**, low acceptance rates, and other indicators of **quality**. It behooves faculty to be aware of the **prestige** rankings of the field's journals; rankings of marketing and out-of-discipline journals are published by the Department and have been distributed to all faculty.

Sentiment: Supportive
Claims to measure: Quality

18.2 Policies and Procedures for Promotion and Tenure (2014), Department of Communication Sciences and Disorders, College of Health & Public Affairs

> ...scholarship in the discipline relates to a range of clinical and research specialties; thus, publication outlets for these specialties include an array of highly respected journals focused in these areas, some of which may include a limited readership due to the narrow focus of the specialty area. Therefore, **it is not possible to interpret such measures as impact factors for these journals in the same manner as it may be possible to interpret them via benchmarks in other fields**. Rather, it is necessary to interpret **impact** and citation **metrics** within the context of the identified specialty areas, which have more narrowly defined readership and scholarly foci. In accordance with the Faculty Senate Resolution on this matter, **"if citation indices are used** relative to the faculty or the institution of the University of Central Florida, **their limitations will be addressed..."**

Sentiment: Cautious
Claims to measure: Unspecified

19 University of Central Florida (continued)

18.3 Criteria for Promotion and Tenure (2014), School of Social Work, College of Health & Public Affairs

> Publications in refereed journals (paper or electronic). **Journal impact factors will not be a primary criteria for the measurement of scholarly activity and prominence** as the academic depth and breadth of the profession requires publication in a multitude of journals that may not have high **impact factors**, especially when compared to the stem [sidisciplines.

Sentiment: Cautious
Claims to measure: Unspecified

18.4 Department Criteria for Tenure and Promotion (2016), Department of Anthropology, College of Sciences

> Evidence of the **impact** of the candidate's work is more important than the sheer number of publications. Scholarly **impact** is indicated by various publication metrics and other recognitions. Publication metrics may include such examples as citation count, **journal impact factor**, journal rankings, journal acceptance rates, journal distribution (international), and the extent of full-text publication downloads. Works that are widely read and cited are generally more **significant** in promotion decisions than obscure, unread and uncited work. In addition, publication in respected, highly cited journals will be given more weight than publications in unranked journals.

Sentiment: Supportive
Claims to measure: Impact/importance/significance

19 University of Central Florida (continued)

18.5 Guidelines for Promotion and Tenure (2012), Department of Biology, College of Sciences

> Research. Requires a sustained record of an internationally recognized research program. This may be demonstrated through:
> a) Publishing in journals judged to be high **quality** as determined by **ISI impact factors**.
> b) Authoring or editing volumes of research in internationally **significant** publications
> c) Having the **importance** of one's research recognized as indicated by citations from other researchers
> ...

Sentiment: Supportive
Claims to measure: Quality

18.6 Departmental Criteria for Tenure and Promotion (2011), Department of Political Science, College of Sciences

> The department expects that those moving to senior rank will have demonstrated a record of **quality** research, and that this research will have earned recognition within the appropriate national scholarly community. Faculty members must document indicators of **quality** of their research and research outlets, such as, but not limited to, **journal impact factors**, journal rankings, citation counts of published work, or acceptance rates.

Sentiment: Supportive
Claims to measure: Quality

19 University of Central Florida (continued)

18.7 Department Criteria for Tenure and Promotion (2015), Department of Sociology, College of Sciences

> It is also true that some refereed journal outlets count for more than others. Publication in respected, highly cited journals, that is, counts for more than publication in unranked journals. The top journals in sociology and all other social sciences are ranked in the Thompson/ISI citation data base (which generates the well-known **Impact Factors**), in the Scopus data base, and in certain other citation data bases. In general, it behooves faculty to be aware of the **prestige** rankings of the field's journals and to publish in the highest-ranked journals possible. It is also advisable to include in one's tenure and promotion file information about the **Impact Factors** or related metrics for the journals where one's papers appear.

Sentiment: Supportive
Claims to measure: Prestige/reputation/status

18.8 Promotion and Tenure Guidelines (2013), Department of Statistics, College of Sciences

> The most common outlet for scholarly research in statistics is in journal articles appearing in refereed publications. Based on the five-year **Impact Factor** (IF) from the ISI Web of Knowledge Journal Citation Reports, the top 50 journals in Probability and Statistics are: [table of ranked journals]

Sentiment: Supportive
Claims to measure: Unspecified

19 University of Guelph

19.1 Department Guidelines Document for Tenure, Promotion, and Performance Assessment (2012), Department of Psychology, College of Social & Applied Human Sciences

> "In evaluating a research publication, the committee will take into account factors such as the scholarly content of the publication, the nature of the review process, the overall scope of the publication, and the **quality** of the journal (**impact indices** may be supplied by faculty)."

Sentiment: Supportive
Claims to measure: Quality

20 University of Guelph (continued)

19.2 Guidelines for Tenure, Promotion and Performance Assessment (2008), Department of Human Health and Nutritional Sciences

> “
> a) Publications
> This will include the **quality** and number of peer-reviewed publications. A faculty member may want to point out the specific qualities of a paper by submitting additional material such as reviewer comments or other documentation. Additionally, the faculty member may provide a few sentences describing the **significance** of these papers and the original contribution that they represent (similar to NSERC F100). Discussion of journal **quality** (by those familiar with the field) may be included in the assessment in addition to consideration of the **quality** of individual research contributions. However, citation analyses and **impact factors** are **problematic indices, particularly in comparisons across fields, and their use in the review process is not encouraged**.
> ”

Sentiment: Cautious
Claims to measure: Unspecified

20 University of La Verne

20.1 Policies and Procedures on Annual, Probationary Status, Promotion, and Tenure Reviews (2016), Department of Psychology

> Each faculty member may choose in the summary narrative to comment on his/her work and how these factors are relevant to his/her research program. In evaluating scholarship, the Committee may also consider the following factors: (a) authorship order/reprint addressee, (b) number of citations, (c) **quality** of the journal (**prestige** of the journal, **impact factor of journal**), and (d) whether the work was conducted after appointment at La Verne.

Sentiment: Supportive
Claims to measure: Quality

21 University of North Carolina at Chapel Hill

21.1 Policy Manual for Faculty Appointments, Promotions, and Tenure, School of Education

> Examples of Standards for Evaluating Research and Scholarship
> ...
> - Publications in peer-reviewed, high **quality**, **high impact journals**. Book and monograph publishers have strong **reputations**. Positive published evaluations of the research/scholarship are available.

Sentiment: Supportive
Claims to measure: Unspecified

21.2 Guidelines for Appointment, Reappointment and Promotion of Faculty (2014), School of Medicine

> Specific criteria for faculty members being promoted to associate professor with tenure on the basis of excellence in research include:
> ...
> 2. A record of a substantial number of original, peer-reviewed research papers in widely respected refereed journals, judged on the **quality** as well as the quantity of research publications, since the faculty member became an assistant professor. Typically 1–2 publications on average per year as first or senior author since the candidate became an assistant professor is expected, although consideration is also given to the type of research, the **impact factor** of the publications, and to faculty whose work is primarily part of team research.

[Note: There are 5 repeat quotes under different promotion levels and areas of scholarship.]

Sentiment: Supportive
Claims to measure: Unspecified

22 University of North Carolina at Chapel Hill (continued)

21.3 Personnel Promotion and Tenure Procedures (2013), School of Social Work

> Table 1. Research and Scholarship
> Criteria:...5. Disseminates scholarship
> Evidence (Supporting Documents)
> ...
>
> - Demonstrates evidence of scholarship **impact**: citation counts, downloads, **impact factors for journals** used

Sentiment: Supportive
Claims to measure: Impact/importance/significance

22 University of North Carolina at Greensboro

22.1 Best Practices in Tenure and Promotion (2016), College of Arts & Sciences

> The evaluation of research should include an explanation of practices and conventions of publishing or exhibiting in the discipline that may not be clear to outsiders...In addition, information on the following points is very helpful:
>
> - The **prestige** and selectivity of the venues in which the candidate has published, performed, or exhibited. **Impact factors** or citation analyses may be included but are not required since such measures are not available in many disciplines. If acceptance rates are available for journals or presses, those should be included.

Sentiment: Supportive
Claims to measure: Prestige/reputation/status

22.2 Promotion and Tenure Guidelines (2013), Department of Nanoscience, Joint School of Nanoscience and Nanoengineering

> Evidence of recognition of research **impact** can also include FSPI [Faculty Scholarly Productivity Index] and media coverage, particularly if in a professional periodical or a leading science journal (e.g., “News and Views" type of highlighting in a major journal). **Impact measures** such as citation data can also be useful for documenting **impact**, but a low citation index should not be used to disallow a promotion, especially if it can be argued that the research challenges a ruling paradigm.

Sentiment: Supportive
Claims to measure: Impact/importance/significance

23 University of North Carolina at Greensboro (continued)

22.3 General Evaluation Guidelines for Reappointment, Promotion and Tenure, and Post-Tenure Review (2014), Department of Nutrition, School of Health and Human Sciences

> "The successful candidate will demonstrate scholarly or creative contributions in a combination of the major areas listed below.
> - Papers in refereed journals (also state **impact factor**, percent acceptance, and circulation volume of each journal)
>
> ..."

Sentiment: Supportive
Claims to measure: Unspecified

23 University of North Dakota

23.1 Faculty Handbook (2008), Department of Biology

> "Exceptional achievement associated with promotion to full professor would be seen in faculty members with several refereed publications each year in national and international outlets, a high proportion of publications in **high impact journals** addressing broad questions in the field of interest, several funded external grants, election to fellow status in national organizations, and/or appointment to a **prestigious** journal's editorial board."

Sentiment: Supportive
Claims to measure: Unspecified

24 University of Saskatchewan

24.1 Standards and Criteria Used by the Food and Bioproduct Sciences Departmental Salary Committee (DSC) to Award Special Increases (SI) to Faculty Members (2012), Department of Food & Bioproducts, College of Agriculture & Bioresources

> “Both the **quality** and quantity of research and scholarly work will be considered in evaluating whether a faculty member’s performance is superior...The faculty member will provide clear documentation of his or her productivity if a case is being made for an SI [Special Increase] under this category. This may include a statement on the **significance** of the work, contributions to multi-authored publications, **impact factors of the journals**, H-factors, etc.”

Sentiment: Supportive
Claims to measure: Unspecified

24.2 Standards for Promotion and Tenure, College of Medicine (2011)

> “The candidate’s published work will be evaluated by assessing the **impact** of the work using a series of **metrics** as identified in Section E. Process of Evaluation. The relative ranking of the publication venue and the number (and sources) of citations in the published works of others are examples of the metrics that may be used.”

[Note: The same quote is repeated three additional times in different parts of the document.]

Sentiment: Not applicable
Claims to measure: Not applicable

25 University of Saskatchewan (continued)

24.3 Guidelines for Preparation of Case Files for Renewal of Probation, Tenure and Promotion (2016)

> Each article, within the review period, should be assessed and reported on for the areas below.
> ...
> v. **Journal impact factor** and disciplinary ranking (both are important, the top Political Science journal has a ranking of 2 while the top science biotech journal has a rating of 25)

Sentiment: Supportive
Claims to measure: Unspecified

24.4 Criteria and Standards (2003), College of Arts and Science, Department of Physics and Engineering Physics

> The publication of research in internationally recognized refereed journals is one of the main criteria used in evaluating a faculty member's performance in research. **Factors** such as the scope and **impact** of the article, and the journal in which it is published will be considered. Candidates may also use citation information to support their case.

Sentiment: Not applicable
Claims to measure: Not applicable

25 University of Southern Mississippi

25.1 Department Guidelines for Tenure and Promotion (2016), Department of Political Science, International Development, and International Affairs

> “While ability and potential as a researcher cannot be inferred from quantitative criteria of publications alone, the following relative weights for types of publications are generally applicable:
>
> ...
>
> f. Consideration will be given to publication **quality** as measured by the following items (though not exclusive of other **quality** measures not listed here): journal/press rankings, journal/press **reputation** in the field, **journal impact factors**, journal acceptance rates, awards, citations, reviews and/or reprints. ”

Sentiment: Supportive
Claims to measure: Quality

25.2 Promotion and Tenure Guidelines (2011), Department of Educational Studies and Research

> “Generally accepted criteria for judging publication or journal **quality** is, foremost, the peer review process and also may include, for example, acceptance rate and **impact factor**. ”

Sentiment: Supportive
Claims to measure: Quality

26 University of Southern Mississippi (continued)

25.3 Revision to Tenure and Promotion Documentation (2015)

> For refereed or peer-reviewed publications, either the acceptance percentage for the journal or other form of publication or the **journal impact factor** (available through Google Scholar - scholar.google.com > Metrics) must be provided. Additional categories such as citations per year, total citations, etc., are encouraged but not required.

Sentiment: Supportive
Claims to measure: Unspecified

25.4 Promotion and Tenure Standards, Department of Philosophy and Religion, University of Southern Mississippi

> The candidate's scholarly contributions should be substantially greater than those that served as the basis for promotion to Associate Professor. However, our primary concern is with assessing the **quality** of the work. At this level we are looking for the kind of contribution that makes a notable positive **impact** on the field. **Factors** described in the accounts of **quality** assessment in I.B. are relevant. Evidence of scholarly **impact** and **quality** can also include favorable reviews of the book, references to the candidate's work by other scholars, and inclusion of the work in anthologies or textbooks. It should also include positive assessments of published work by respected scholars with expertise in the candidate's area from other universities.

Sentiment: Not applicable
Claims to measure: Not applicable

26 University of Utah

26.1 Policies and Procedures for Retention, Promotion, and Tenure of Regular Faculty (2012), Department Of Political Science, College of Social & Behavioural Science

> “**Quality** is the extent to which the research is consistent with the methods and goals of the field, shaped by knowledge that is current and appropriate to the topic, and well written. **Quality** is best measured by experts in the field, including peer reviewers for publications, external reviewers solicited for the RPT review, and University of Utah colleagues who have personally read the publications. In the discipline of political science the **reputation** of various scholarly outlets is a valid surrogate measure of the **quality** of the scholarship published therein...Candidates are expected to cite evidence to support claims about the **quality** of publication outlets. Candidates are encouraged to use evidence such as journal or publisher rankings, **journal impact scores**, other books in a publisher’s list, and/or editorial board composition. Electronic publications count the same as traditional print publications if these indicators of **quality** are comparable.”

Sentiment: Supportive
Claims to measure: Quality

27 University of Utah (continued)

26.2 Policies and Procedures for Retention, Promotion, and Tenure Procedures and Criteria (2011), Department of Sociology, College of Social & Behavioral Sciences

> Peer reviewed research provides the most convincing evidence of research excellence, and the more esteemed the outlet for a publication, the greater the **impact** it is likely to have on the research literature. In research/scholarship, some Candidates may pursue a broad number of areas; others may focus on a single topic. In either strategy, however, high **quality** demands that the work show thoroughness, adhere to relevant standards of rigor and research **quality**, and other evidence of excellence, as described above. Peer reviewed publications in highly regarded journals (general sociology, interdisciplinary, and subfield) indicate excellence as do books with well regarded University presses. The publication portfolio should include work in journals with high **impact factors** within the Candidates' fields of research.

Sentiment: Supportive
Claims to measure: Unspecified

27 University of Vermont

27.1 Guidelines for Reappointment, Promotion, and Tenure (2009), College of Agriculture and Life Sciences

> The essential elements of the dossier are:
> ...
> - a description of the **impact** or **prestige** of each scholarly outlet; that is, an indication to the evaluator regarding the tier **significance** of the journal, professional meeting, readership scope of the book or book chapter, or **impact factor**. A short summary of the **impact** provided by the candidate's most **significant** scholarly outputs may be included.

[Note: This (or similar) quote appears 3 more times in the document under different sections.]

Sentiment: Supportive
Claims to measure: Prestige/reputation/status and impact/importance/significance

27.2 Statement Regarding the Appointment, Tenure, and Promotion of Tenure-Track Faculty (2015), College of Arts and Sciences

> The evaluation of **quality** normally consists of an assessment of the originality, skill, and **importance** of the candidate's contribution to the field. **Quality** is assessed through unbiased, expert analysis and judgment (e.g., internal and external peer evaluation, book reviews, etc.), as well as inferred through indicators such as the **prestige** and **reputation** of publication outlets, contextualized **impact factors**, and the selectivity of the journals, presses, galleries, venues, etc. where the work appears.

Sentiment: Supportive
Claims to measure: Quality

28 University of Vermont (continued)

27.3 Guidelines for the C.V. for Green Sheets (2013)

> The **status** of publication venue (e.g., selectivity) must be addressed on the green sheet form. Inclusion of **impact factor** and citation index data is helpful where available.

Sentiment: Supportive
Claims to measure: Prestige/reputation/status

27.4 Reappointment, Promotion and Tenure (RPT) Guidelines and Forms, Green Sheet Form (2016)

> List all works reviewed prior to publication by peers / editorial boards in the field, such as journal articles in refereed journals, juried presentations, books, etc. Indicate up to five of the most **important** contributions with a double asterisk and briefly explain why these choices have been made. Include a description of the **stature** of journals and other scholarly venues and how this is known (e.g., **impact factors**, percentage of submitted work that is accepted, together with an explanation of the interpretation of these measures).

Sentiment: Supportive
Claims to measure: Prestige/reputation/status

28 University of Victoria

28.1 Faculty Evaluation Policy (2016), Faculty of Science

> “In the Faculty of Science, the criteria for the assessment of Scholarly and Professional Achievement will include, but is not limited to, the following:
>
> - authorship of refereed research publications in recognized scholarly journals where the expectation for the number of papers published will be appropriate to the discipline...Members are invited to explain their choice of journals. This may be done by making reference to **journal impact factors** (if so desired) or by explaining the **importance** of the journal to their discipline in their Research Statement
>
> ...”

Sentiment: Supportive
Claims to measure: Unspecified

29 University of Windsor

29.1 Standards: Example of multiple minimum thresholds in one document (2016)

> Criterion 3: Evidence of independent and original contributions to research or creative activity, which have an **impact** on the field of expertise.
>
> Associate Professor (Level A)
> Evidence of original contributions that are influencing the evolution of the field, practice, or thinking within the discipline or as practical applications.[2]
>
> [2]Some departments may wish to provide more specific quantifiers based on factors including **impact factors of journals**, citation counts, and elements of the candidate's research statement supported by evidence, or alternatively to include examples such as patents, policy contributions, etc. Some departments also combine **impact** and publication record, depending on the nature of the discipline.

[The first part of this quote (up to 'evidence') also appears in the Sample Research Evaluation Rubric, and appears in its entirety in the Working Session on Developing Promotion and Tenure Criteria for Research (2016) document from University of Windsor.]

Sentiment: Supportive
Claims to measure: Impact/importance/significance

30 University of Windsor (continued)

29.2 Sample Research Evaluation Rubric (2016)

> Criterion 2: A record of high **quality** refereed publications, juried creative activity or other demonstrated scholarly outputs
>
> a) Publishes in journals or with publishing houses with a strong academic **reputation**[2]
>
> [2]Departments may wish to provide quantitative metrics such as **journal impact factors** as an element of their standards. Factors such as low acceptance rates, high levels of readership, **importance** to the field are also suggestive indicators in assessing **quality** and **reputation**. Some departments have used an illustrative model to articulate publications that are at acceptable levels. Departments may also wish to identify the range of publications or other products that should be taken into account.

[Note: The first part of this quote (up to 'reputation') also appears in the Working Session on Developing Promotion and Tenure Criteria for Research (2016) from University of Windsor.]

Sentiment: Supportive
Claims to measure: Quality and prestige/reputation/status

> Criterion 3: Evidence of independent and original contributions to research or creative activity, which have an **impact** on the field of expertise.
>
> a) Original contributions to the field of study or creative practice that influenced thinking and/or practice in the field, including extent to which research or creative activity is considered, referred to, read; citation in documents; **impact factors**, citation counts, publication rates, confidential external reviews of **impact**

[Note: A nearly identical quote also appears in the Working Session on Developing Promotion and Tenure Criteria for Research (2016) and the Research Evaluation Framework (2016) documents, both from the University of Windsor]

Sentiment: Supportive
Claims to measure: Impact/importance/significance

30 University of Windsor (continued)

29.3 Working Session on Developing Promotion and Tenure Criteria for Research (2016)

> The Department acknowledges that journals and books vary in **quality**. At the same time, the Department recognizes that it is difficult to measure journals' or books' worth by using **impact factors** or other similar indicators. Generally, books published by university presses and journals linked to scholarly associations or published by well-recognized publishers (e.g. Taylor and Francis, Routledge, Sage, etc.) will be assigned greater value than other publications. Candidates will be encouraged to submit a statement that explains the **importance** of their publications, which may include factors such as **journal impact factors**, citation rates, publication in journals with low acceptance rates, high levels of readership, demonstrated **importance** to their field.

Sentiment: Supportive
Claims to measure: Impact/importance/significance

> Publications and contribution are in well reputed and appropriate venues with **significant impact** in her research area. This may include community, local, national and international venues that are highly regarded and of **significance** to the field. It may include both contributions in traditional and non-traditional venues and both publicly engaged and peer reviewed contributions.
>
> The extent to which the publications influence the field based on number of citations, citation rate, **impact factor**, confidential reviews, invitation as keynote speaker or peer reviewer.

Sentiment: Supportive
Claims to measure: Impact/importance/significance

30 University of Wisconsin-Madison

30.1 Guidelines for Recommendations for Promotion or Appointment to Tenure (2014), Faculty Division of the Physical Sciences

> List of research publications...Provide acceptance rates and/or **impact factors** if available...
> Please list publications of different types separately in the following format:
> a. Papers published in, or accepted by, refereed archival publication venues (e.g., journals and highly selective conferences with archival proceedings). Papers must be accessible for reference. Comment on the standards of the publication venue, and provide a list of the top (not more than 10) publication venues in the candidate's research areas, identifying both broad and specialty venues. Include acceptance rates and/or **impact factors** where available (be sure to specify the research areas), and provide in an appendix a copy of the letter of acceptance if not yet published. Citation counts and/or electronic download counts may be included to document **impact**.

Sentiment: Supportive
Claims to measure: Unspecified

30.2 Format for Tenure Dossiers submitted to the Physical Sciences Divisional Committee, Checklist for Departments (2014)

> List of research publications:
> a) Papers in refereed archival publication venues; publication venue rankings, standards, acceptance rates and/or **impact factors**. Citation counts and/or electronic download counts optional.

Sentiment: Supportive
Claims to measure: Unspecified

31 University of Wisconsin-Madison (continued)

30.3 Getting Promoted From Assistant to Associate Professor on the Tenure Track (2015), Faculty Division of Biological Sciences, Department of Radiology

> The faculty member is expected to have multiple publications during the probationary period, with evidence for increasing productivity and **impact** of the publications during the probationary period. The publications should be in **high impact journals** such as Radiology, AJR, or the leading subspecialty journals in the candidate's field and should generally be focused on the candidate's research topic.

Sentiment: Supportive
Claims to measure: Unspecified

> Scenario 1 Investigator initiated research
> Principal Investigator on NIH R01 (or equivalent)
> 15 publications during the probationary period while at UW (at least 10 as corresponding author – first or last author). The publications should be in **high impact journals** and at least 5 should be focused on the candidate's research topic.

[Note: A nearly identical quote is found in the same document under Scenario 2 Team Research for Co-PI's.]

Sentiment: Supportive
Claims to measure: Unspecified
